# Woody Plant Encroachment into Grasslands: Spatial Patterns of Functional Group Distribution and Community Development

**DOI:** 10.1371/journal.pone.0084364

**Published:** 2013-12-18

**Authors:** Feng Liu, Steven R. Archer, Frances Gelwick, Edith Bai, Thomas W. Boutton, Xinyuan Ben Wu

**Affiliations:** 1 Key Laboratory of Aquatic Botany and Watershed Ecology, Wuhan Botanical Garden, Chinese Academy of Sciences, Wuhan, China; 2 Department of Ecosystem Science and Management, Texas A&M University, College Station, Texas, United States of America; 3 School of Natural Resources and the Environment, University of Arizona, Tucson, Arizona, United States of America; 4 Department of Wildlife and Fisheries Sciences, Texas A&M University, College Station, Texas, United States of America; 5 State Key Laboratory of Forest and Soil Ecology, Institute of Applied Ecology, Chinese Academy of Sciences, Shenyang, China; Beijing Forestry University, China

## Abstract

Woody plant encroachment into grasslands has been globally widespread. The woody species invading grasslands represent a variety of contrasting plant functional groups and growth forms. Are some woody plant functional types (PFTs) better suited to invade grasslands than others? To what extent do local patterns of distribution and abundance of woody PFTs invading grasslands reflect intrinsic topoedaphic properties versus plant-induced changes in soil properties? We addressed these questions in the Southern Great Plains, United States at a subtropical grassland known to have been encroached upon by woody species over the past 50-100 years. A total of 20 woody species (9 tree-statured; 11 shrub-statured) were encountered along a transect extending from an upland into a playa basin. About half of the encroaching woody plants were potential N_2_-fixers (55% of species), but they contributed only 7% to 16 % of the total basal area. Most species and the PFTs they represent were ubiquitously distributed along the topoedaphic gradient, but with varying abundances. Overstory-understory comparisons suggest that while future species composition of these woody communities is likely to change, PFT composition is not. Canonical correspondence analysis (CCA) ordination and variance partitioning (Partial CCA) indicated that woody species and PFT composition in developing woody communities was primarily influenced by intrinsic landscape location variables (e.g., soil texture) and secondarily by plant-induced changes in soil organic carbon and total nitrogen content. The ubiquitous distribution of species and PFTs suggests that woody plants are generally well-suited to a broad range of grassland topoedaphic settings. However, here we only examined categorical and non-quantitative functional traits. Although intrinsic soil properties exerted more control over the floristics of grassland-to-woodland succession did plant modifications of soil carbon and nitrogen concentrations, the latter are likely to influence productivity and nutrient cycling and may, over longer time-frames, feed back to influence PFT distributions.

## Introduction

Increases in the abundance of trees and shrubs have been reported in ecosystems world-wide [1-3]. The resultant change in physiognomy from grassland, steppe or savanna to shrubland or woodland has significant impacts on ecosystem productivity, trophic structure, nutrient cycling and biodiversity [4-8], and may have significant implications for local [9], regional [10,11] and global [12] carbon cycling given the geographic extent of these ecosystems on Earth. Woody plant proliferation in recent decades has been reported in arctic tundra, in temperate, subtropical, tropical, coastal and montane grasslands, in hot and cold desert grasslands, and in savannas and steppe [3,4,13,14]. Because woody plant encroachment is a worldwide phenomenon, it is important to understand it in general terms if we are to anticipate and predict where and how the abundance of shrubs and trees might change under current and future environmental conditions. To date, although there are studies focused on multiple invaded woody species [15-17], the majority of studies related to woody plant encroachment have focused on key encroaching species [11]. From such studies we can only speculate as to i) which of the many traits specific to those species make them so successful; and ii) the relative importance of those traits.

A qualitative survey of published literature indicates that encroaching woody species represent a broad range of plant functional types (PFTs), ranging from shrub to tree in stature; from evergreen to deciduous, malacophyllous to sclerophyllous, and broad-leaved to needle-leaved in leaf habit, structure and size; from N_2_-fixing to non-fixing; from deep rooted to shallow rooted; and from mesophytic to xerophytic in water relations [18-22]. However, robust generalizations regarding traits or suites of traits that may make some species and the PFTs they represent more successful than others in invading grasslands do not exist. Are some woody plant growth forms and PFTs better suited than others to invade grasslands; and if so, under what environmental conditions? Furthermore, it is well-known that woody plants may modify soils and microclimate subsequent to their establishment. To what extent does this induced change affect woody PFT distribution and abundance?

**Figure 1:**
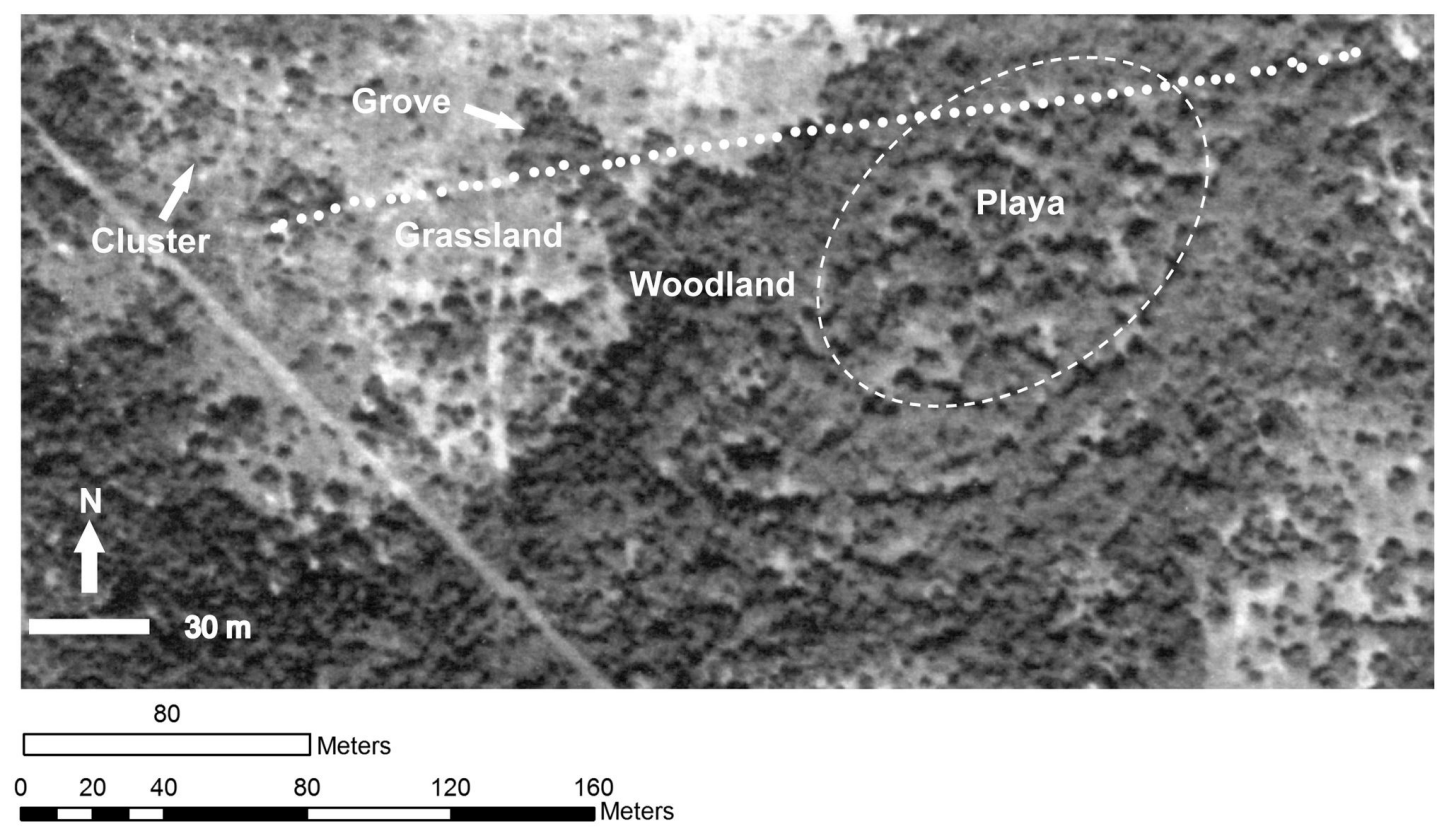
Aerial view of the study site showing a transect spanning shrub cluster and grove communities in savanna parkland uplands, a woodland community in an intermittent drainage, and a playa savanna community. Each of these communities is developing on former grassland. White dots are GPS locations at 5 m intervals. Dark and light gray colors indicate woody and herbaceous vegetation, respectively. Trees and shrubs were inventoried in contiguous plots (6 x 6 m and 2 x 2 m, respectively) along the transect.

The Southern Great Plains of North America is a region where the conversion of grass to woody plant dominance over the past 100+ years has been well-documented by a variety of sources including diaries of early settlers, time-series remote sensing, tree rings, carbon isotopes, and ecosystem models [23]. Potential drivers of this shift in life form abundances include livestock grazing, fire suppression, climate change, and atmospheric CO_2_ enrichment [24]. Topoedaphic properties [25] and fluctuations in native herbivore abundance [26] may locally constrain or mitigate responses to these drivers. While the phenomenon of woody plant encroachment and its effects on soil C and N cycles has been widely examined in this region [9], details pertaining to the development of woody plant communities in contrasting topoedaphic settings are largely unknown. The woody flora of the subtropical Tamaulipan Biotic Province, which encompasses southern Texas and northern Mexico [27], is highly diverse [18] and includes an array of PFTs [28]. This high floristic diversity of woody vegetation affords the opportunity to determine if certain woody PFTs might be better suited than others for encroachment into grasslands. Here, we sought to determine similarities and differences in the PFT composition of four shrub communities with varying establishment time developing on former grasslands along a landscape-scale catena (hill-slope) topoedaphic gradient (Figure 1). Specifically, we asked: (1) What are the similarities and differences in species and woody PFT composition of these communities? (2) Do PFT overstory-understory relationships change along the gradient? and (3) What is the influence of intrinsic soil physical properties (e.g. texture) relative to that of plant-induced changes in soil chemical properties (e.g., organic carbon and nitrogen content) on PFT abundance and distribution? Because our study was conducted on a local scale we were able to control for climate and land use history, and factors that might otherwise confound comparisons of species and functional types.

## Methods

### Study site

Field studies were conducted at the Texas AgriLife La Copita Research Area (LCRA, 27°40’N; 98°12’W ), approximately 24 km southwest of Alice, Texas, USA. LCRA is owned by Texas A&M University and no specific permission is required to do research at this site. This study did not involve any endangered or protected species. Climate of the region is subtropical with a mean annual temperature of 22.4°C and mean annual precipitation of 680 mm. Annual precipitation is bi-modally distributed with maxima in May/June and September. Elevation ranges from 75 to 90 m. The site was a working cattle ranch with a long history of livestock grazing before its designation as a research area in early 1980s. Fire has been suppressed while it was a working ranch and later as a research site. In the 1980s and 1990s, various brush management practices (mechanical and herbicidal) were implemented on the site. The woody plant flora at this site consists of 49 species [19] encompassing a range of stature and ‘woodiness’ that ranges from trees (arboreal) to shrubs (fruticose) to subshrubs (suffruticose/suffrutescent); and a wide range of leaf traits (texture, longevity, specific leaf area, N content) and rooting depths [20,21]. Gas exchange, water relations and isotopic composition of the various plant functional types (PFTs) have been well-described [29-33].

Uplands with sandy loam surface soils (Typic Argiustolls) are characterized by savanna parklands (Fig. 1). A well-developed argillic horizon ~ 40 cm below the surface is laterally extensive and supports a grassy matrix containing scattered, discrete shrub clusters. Cambic inclusions with poorly expressed argillic horizons are dispersed throughout the uplands and support large groves of woody vegetation [1]. Convex uplands grade gently (1-3% slopes) into lower-lying intermittent drainages with clay loams and clays (Pachic Argiustolls) characterized by closed-canopy woodlands. Oval-shaped playas (Ustic Epiaquerts and Vertic Argiaquolls) with no external drainage occupy the lowest portions of the landscape, with vegetation ranging from grassland to open woodland [34,35]. *Prosopis glandulosa* var. *glandulosa* (hereafter “*Prosopis*”) is the dominant overstory in all woody communities. *Zanthoxylum fagara , Condalia hookerii, Diospyros texana, Celtis pallida*, and *Berberis trifoliolata* are common shrub species (plant nomenclature follows [36]). Upland grasslands consist primarily of C_4_ grasses in the genera of *Aristida*, *Bouteloua*, *Cenchrus*, *Chloris*, and *Setaria*; and a diversity of herbaceous dicots. Playa communities are typically characterized by a dense and continuous ground layer of the C_4_ grasses *Paspalum pubiflorum* var. *pubiflorum* and *Bothriochloa ischaemum* [34].

### Field sampling and lab analyses

A 309-m transect traversing the major plant communities (grassland, discrete cluster, grove, drainage woodland, and playa) was established along the topoedaphic gradient extending from the convex upland downslope into the concave playa basin (Figure 1). Elevation was determined based on a topographic field survey conducted in April 2004.

Soils cores (15 cm deep, 2.24 cm diameter) were collected at 1 m intervals (2 cores per point) along the transect. One core was used to quantify bulk density (BD) and soil texture, and the other was used to determine soil organic carbon (SOC), total nitrogen (TN), and pH. Another set of soil samples was collected at 3-m intervals during a 2-day period (no precipitation either day) in April 2005 to determine soil volumetric water content (VWC). We recognize that the soil VWC is highly dynamic; and that a one-time measurement of soil VWC at a single time and shallow depth is highly superficial. Thus, we used VWC only as a rough indicator of potential spatial variation in moisture along the catena gradient. Woody species with few and relatively large (basal diameter > 5 cm) stems and a height potential of > 4 m were recorded as trees. Their basal diameters were measured in contiguous 6 m × 6 m cells (plots) centered along the transect (n=51). The size and abundance of shrubs with relatively small stems (basal diameter < 5 cm) was recorded within 2 m × 2 m cells (plots) centered on the soil sample points (n = 154) along the transect. The shrub category thus included juveniles of woody species with potentially arborescent stature. Species characterized by suffrutescent growth habits (herbaceous perennials with woody bases) were not included. 

We characterized woody species into different functional types according to available references and field observations. N_2_-fixing PFT data is from Zizter et al. 1996 [22]; Leaf texture data is from Nelson et al. 2002 [21]; root depth information is from Watts 1993 [20] and Boutton et al. 1999 [37].

Soil VWC was determined by weighing soil cores before and after oven drying at 105°C for 24 hours. Bulk density and soil texture were quantified using the core method and pipet method respectively [38]. Soil cores designated for SOC and TN determination were dried at 60°C for at least 48 hours, passed through a 2 mm screen to remove coarse organic fragments and gravel, and then pulverized to a fine powder in a centrifugal mill (Angstrom, Inc., Belleville, MI, USA). Samples were weighed into silver capsules (5 x 7 mm) using a microbalance, treated with HCl vapor in a desiccator to volatilize carbonate-carbon, dried thoroughly, and sealed into the capsules. SOC and TN concentrations were determined by dry combustion using an elemental analyzer (Carlo Erba EA-1108, CE Elantech, Lakewood, NJ). The concentrations were then converted to densities (g m^-2^) to a depth of 15 cm by multiplying by BD. Soil pH (Accumet Basic pH meter, Fisher Scientific) was determined using a CaCl_2_ solution (0.01 M CaCl_2_) containing 12 g soil. 

### Statistical analyses

Soil variables in each plant community type were compared using ANOVA with Tukey’s corrections for *post hoc* comparisons. Shrub and tree distributions along the topoedaphic gradient were explored using canonical correspondence analysis (CCA; [39]) in PC-ORD (version 5, MjM software, Gleneden Beach, OR; [40]). CCA was performed on two matrices: the plot by species matrix as the dependent variables, and the plot by environmental variables matrix as the independent (explanatory) variables. Environmental variables included soil texture (% sand, % clay), SOC, TN, pH, and BD. Silt was excluded because it is a linear combination of sand and clay (silt=100 – sand – clay), and VWC was excluded owing to our low sampling frequency. Matrices of total basal area per plot were analyzed separately for trees and shrubs because their plot sizes differed (6 m × 6 m and 2 m × 2 m, respectively). Environmental variables for each plot in the matrix were averages of their respective within-plot replicate measurements. 

Partial CCA in PC-ORD was used to partition the explained variation of woody species composition and to quantify the relative influence of environmental variable groups on community structure. Inertia of ordination, which quantifies variation in species composition, is additive and can be distributed into groups of environmental variables [39]. Here, we used the proportion of total variation explained (TVE) by groups of environmental variables instead to quantify their relative contributions [41,42]. The relative influence of two groups of environmental variables was evaluated for the tree and the shrub species matrices. Prior to partial CCA, variables in each group were evaluated for their independent and significant contribution to variation in species composition [43] using randomization tests. Only significant (p < 0.05) variables were included in variation partitioning, as described in [44,45] (pH was excluded as it was non-significant). One group of environmental variables was comprised of soil texture (% sand, % clay) reflecting long-term pedogenesis along the hill-slope gradient. The second group of variables included SOC and TN, both of which are known to increase with time of site occupation by woody plants at this site [9,23]. We did not include BD in either group because it represents both long-term pedogenesis and shorter-term effects of organic matter additions and was not a strong explanatory variable 

## Results

### Species composition and soil properties

**Figure 2:**
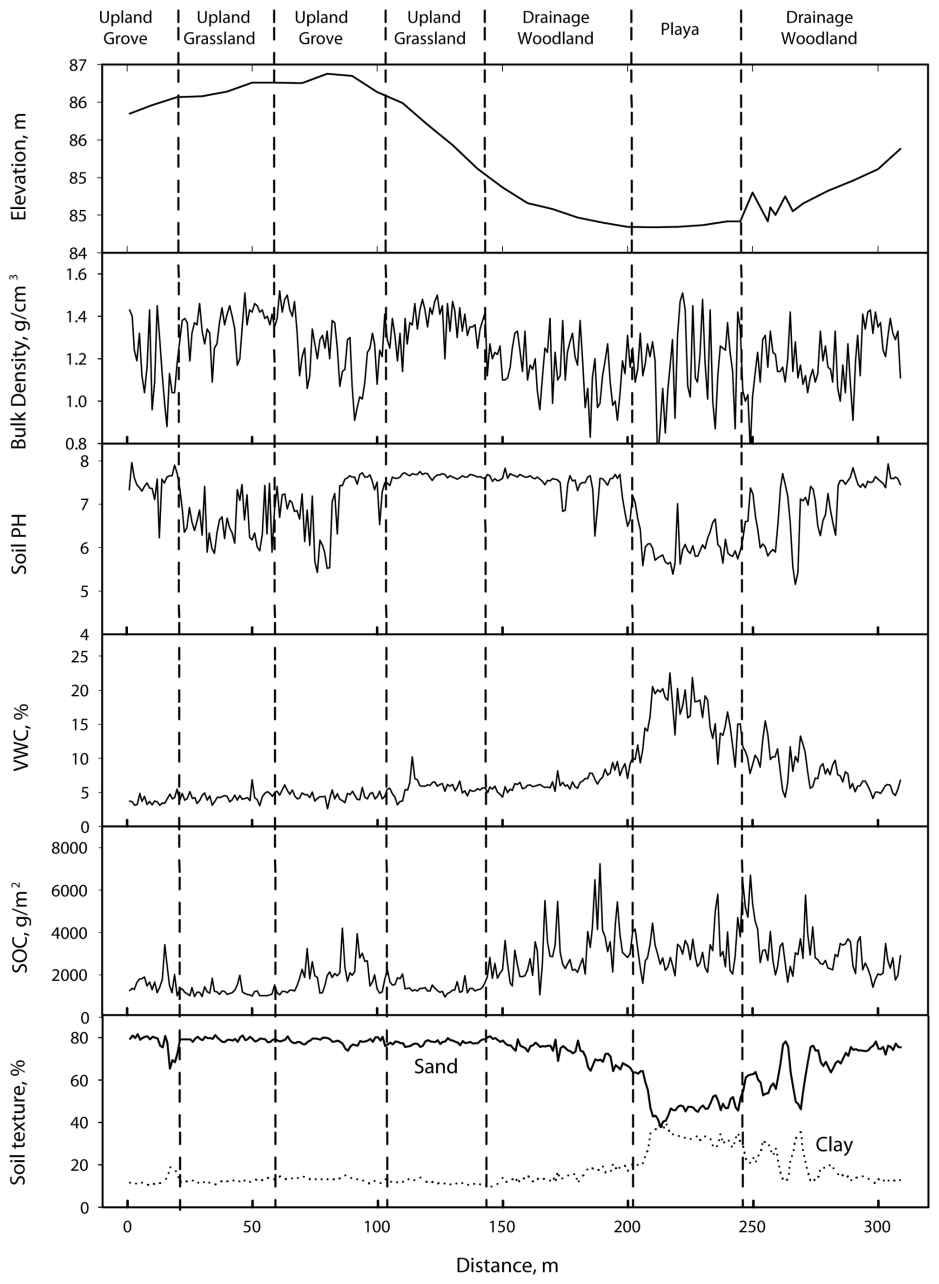
Topoedaphic variations based on sampling at 1-m intervals along a transect extending from plant communities in a savanna parkland upland (grassland, shrub cluster, grove), through intermittent drainage woodlands and into a playa savanna community. VWC = soil volumetric water content; SOC = soil organic carbon.

Changes in elevation and edaphic properties along the hill-slope transect are depicted in Figure 2 and summarized in Table 1. On the date of sampling, soil VWC was highest in playa basins and the woodlands near these basins, and was uniformly lower elsewhere. Soil (0-15 cm) sand content was the greatest in the uplands, with clay content increasing down-slope along the catena and peaking in the playa basin. BD was highly variable along the hillslope. SOC density was also variable, but generally higher in intermittent drainage woodland and playa landscape locations and lower in uplands. Within uplands, the grassland community had uniformly low SOC with distinctive peaks in SOC being associated with grove and shrub cluster communities embedded within the grassland matrix. Spatial patterns of TN mimicked those of SOC (data not shown; correlation between SOC and TN =0.93; p<0.01). 

**Table 1:** Mean (+ standard error) values of soil (0-15 cm depth) variables and ANOVA results along a catena gradient in a subtropical grassland undergoing woody plant encroachment.

	Upland Grassland	Upland Cluster	Upland Grove	Drainage Woodland	Playa	ANOVAp
Bulk density (g cm^-3^)	1.4±0.01**^*a*^**	1.3±0.02^ab^	1.2±0.02**^*b*^**	1.2±0.01**^*b*^**	1.8±0.03**^*b*^**	<0.001
VWC (%)	5.1±0.1**^*a*^**	4.6±0.4**^*a*^**	4.2±0.1**^*a*^**	7.4±0.2**^*b*^**	16.4±0.5**^*c*^**	<0.001
PH	7.1±0.1**^*a*^**	7.1±0.2**^*a*^**	7.2±0.1**^*a*^**	7.2±0.1**^*a*^**	6.0±0.1**^*b*^**	<0.001
Organic carbon (g m^-2^)	1233±22**^*a*^**	1482±69^ab^	1925±87**^*b*^**	3035±99**^*c*^**	3142±155**^*c*^**	<0.001
Total nitrogen (g m^-2^)	123±2**^*a*^**	143±5^ab^	182±8**^*b*^**	288±10**^*c*^**	253±14**^*c*^**	<0.001
Sand (%)	79±0.1**^*a*^**	78±0.4**^*a*^**	78±0.4**^*a*^**	70±0.7**^*b*^**	48±0.8**^*c*^**	<0.001
Silt (%)	9±0.2**^*a*^**	10±0.3**^*a*^**	9±0.2**^*a*^**	13±0.2**^*b*^**	20±0.3**^*c*^**	<0.001
Clay (%)	12±0.1**^*a*^**	12±0.2**^*a*^**	13±0.2**^*a*^**	17±0.5**^*b*^**	32±0.6**^*c*^**	<0.001

Different superscripts indicate significant difference among communities. Number of samples: grassland=66, cluster=18, grove=59, woodland=125, and playa=41.

A total of 9 species with tree-stature potential occurred along the topoedaphic gradient (Table 2). The number of tree species encountered was lower in shrub cluster (4) and playa (6) communities; and higher in grove (7) and drainage woodland (8) communities. *Prosopis* trees in shrub cluster communities were dead, so were not included in these tallies. Among tree species, *Acacia farnesiana* (deciduous, potential N_2_-fixer; note: ‘potential’ is hereafter implicit in all uses of ‘N_2_-fixer’) and *Z. fagara* (evergreen, non-fixer) occurred in all four woody plant communities. *Acacia rigidula* (deciduous; N_2_-fixer) was restricted to woodland communities; and *Parkinsonia aculeata* (deciduous; no known report of N_2_-fixation, thus treated as non-fixer) was restricted to playa communities. *Z. fagara* had the highest stem density (183 ha^-1^), with four other tree species having stem densities >100 ha^-1^. *Prosopis* (deciduous, N_2_-fixer) had the greatest average individual plant stem basal area (543 cm^2^/plant). 

**Table 2:** Occurrence of tree-stature species (> 5 cm basal diameter) within 6 × 6m contiguous plots along a transect spanning shrub cluster and grove communities in savanna parkland uplands, woodlands of intermittent drainages, and a playa (Figure 1).

Species	Cluster		Grove		Woodland		Playa	
	N	BA	N	BA	N	BA	N	BA
*Acacia farnesiana*	2	0.6±0.1	2	0.2±1.4	8	1.6±3.9	19	17.0±3.3
*Acacia rigidula*	0	-	0	-	2	0.5±4.6	0	-
*Celtis pallida*	0	-	5	0.7±0.7	37	3.8±1.0	22	6.6±1.1
*Condalia hookeri*	6	2.5±1.3	5	0.7±2.0	39	2.4±0.6	0	-
*Diospyros texana*	0	-	3	0.3±1.1	50	2.1±0.3	5	0.5±0.2
*Karwinskia humboltiana*	2	0.6±1.8	4	0.3±0.3	1	0.1	0	-
*Parkinsonia aculeata*	0	-	0	-	0	-	1	0.46
*Prosopis glandulosa*	0	-	22	9.6±1.5	34	13.2±1.9	4	12.4±9.8
*Zanthoxylum fagara*	2	4.2±11.7	21	4.2±1.4	37	2.7±0.8	8	1.3±1.5

N = number of plants; BA = basal area (cm^2^ m^-2^; mean ± standard error). *Prosopis glandulosa* var. *glanduloas* was present in shrub clusters, but had died and hence was not recorded. See Table 3 for growth form and functional group categories represented by the species.

A total of 20 shrub species were encountered within the 2-m wide belt centered on the transect line. All species with tree-stature potential (Table 2) also occurred as shrubs (Table 3). Individuals of *A. rigidula* (deciduous, N_2_-fixer)*, Aloysia gratissima* (drought deciduous, non-fixer)*, Bernardia myricaefolia* (deciduous, non-fixer) and *Epheda antisyphilitica* (stem photosynthesis, non-fixer) were encountered only in the woodland community; *A. greggii* (deciduous, N_2_-fixer) occurred only in the discrete cluster community; and shrub-stature *Parkinsonia aculeata* plants (deciduous, non-fixer) occurred only in the playa. In contrast, *Z. fagara* and *C. pallida* shrubs occurred in all four plant communities. *Prosopis*, *D. texana*, *P. aculeata*, and *Acacia spp.* were among the largest shrubs (average basal areas > 6 cm^2^ per stem), and *C. texensis* was among the smallest (average basal area per stem < 0.5 cm^2^). 

**Table 3:** Occurrence (N) and mean basal area (BA, mean + SE; cm^2^ m^-2^ ) of shrub-stature species within a 2-m wide belt transect (Figure 1).

Species	Functional attributes			Cluster		Grove		Woodland		Playa	
	Leaves	N-fix	Roots	N	BA(cm^2^)	N	BA(cm^2^)	N	BA(cm^2^)	N	BA(cm^2^)
*Acacia farnesiana^$^*	*D, M*	*,#	*Deep*	0	-	0	-	2	30.8±1.4	1	22.1
*Acacia greggii^$^*	*D, C*	*,##	*Int*	5	30.7±0.3	0	-	0	-	0	-
*Acacia rigidula^$^*	*D, C*	*,#	*Int*	0	-	0	-	13	111.4±0.5	0	-
*Aloysia gratissima^$^*	*D, M*		*Shallow*	0	-	0	-	257	173.5±0.0	0	-
*Berberis trifoliolata**^§¶^***	*E, S*		*Shallow*	19	31.3±0.2	10	10.9±0.2	20	17.5±0.1	0	-
*Bernardia myricaefolia*	*D, M*		*-*	0	-	0	-	1	0.5	0	-
*Celtis pallida**^§¶^***	*D, C*	*,##	*Int*	6	1.8±0.1	109	530.1±0.1	562	1732.8±0.0	5	96.6±0.7
*Colubrina texensis^$^*	*D, C*	*,##	*Deep*	289	106.7±0.0	313	151.9±0.0	30	22.2±0.1	0	-
*Condalia hookeri**^§^**^$^*	*FE,M*	*,##	*Int*	15	73.4±0.5	18	9.7±0.0	12	187.4±0.7	0	-
*Diospyros texana**^§^**^$^*	*FE,C*		*Int*	0	-	12	28.0±0.3	136	854.6±0.1	7	115.5±1
*Ephedra antisyphilitica*	*D,PS*		*-*	0	-	0	-	1	2.8	0	-
*Eysenhardtia texana*	*D,M*	*,#	*-*	22	50.4±0.2	0	-	0	-	0	-
*Forestiera angustifolia*	*E,M*		*-*	2	3.5±0.3	1	2.8	4	9.1±0.2	0	-
*Gymnosperma* spp.	*D,C*		*-*	0	-	10	4.3±0.1	1	2.3	0	-
*Karwinskia humboltiana*	*E,C*	*,##	*-*	0	-	113	363.1±0.1	184	190.4±0	0	-
*Parkinsonia aculeata*	*D, M,PS*	*	*Shallow*	0	-	0	-	0	-	2	13.2±0.1
*Prosopis glandulosa**^§¶^***	*D,C*	*,#	*Deep*	1	12.6	9	178.9±0.7	10	123.4±0.7	0	-
*Schaefferia cuneifolia**^¶^***	*E,S*		*Int*	20	31.2±0.1	84	58.8±0	160	101.5±0	0	-
*Zanthoxylum fagara**^§¶^***	*E,C*		*Shallow*	85	190.1±0.1	169	574.3±0.1	140	490.6±0.1	3	10.2±0.7
*Ziziphus obtusifolia**^§¶^***	*SD,M*	*,##	*Int*	0	-	3	14.4±0.6	4	11.3±0.4	5	19.6±0.4

Functional attribute codes represent leaf habit, leaf texture, rooting depth and N_2_-fixation potential respectively. Codes are as follows: Leaf habit [E = evergreen, FE = facultative evergreen (leaves maintained during mild winters), D= deciduous, SD=summer deciduous, PS =photosynthetic stem]. Leaf texture [S = sclerophyllous (thick and stiff),C= coriaceous (thick and leathery), M = malacophyllous (thin and pliable)] (based on authors’ field observations or, where noted, Nelson et al. 2002)]. N-fixation [* = potentially N_2_-fixing (based on family affinities; e.g. Fabaceae, Rhamnaceae). ***^#^*** and **^*#*^** *,* ***^#^*** = species for which N_2_-fixation potential has been assessed (Zitzer et al. 1996) and for which nodulation was found to occur (#) or not occur (##)]. Root depth [*int* = intermediate, *Deep*=deep, *Shallow*=shallow]. §,¶ *,*$ = species for which information on leaf attributes (longevity, specific leaf area, N content, etc.; § = from Nelson et al. 2002) and relative rooting depth (¶ = from Watts1993; $ = from Boutton et al. 1999) are known.

*Prosopis* (deciduous, N_2_-fixer) was the dominant overstory plant in all woody plant communities (although it had died in the shrub clusters). *C. texensis* (deciduous, non-fixer) and *Z. fagara* (evergreen, non-fixer) dominated the shrub component in upland clusters and groves, whereas *C. pallida* (deciduous, non-fixer) and *D. texana* (deciduous, non-fixer) dominated the shrub understory in intermittent drainage and playa communities (Table 3). 

Woody plant species richness (maximum encountered) was lowest in playa communities (n=7), highest in woodlands (n=17) and intermediate in shrub clusters and groves (n= 12 and 13, respectively) (Table 4A). Richness of deciduous species in lowland woodland and playa communities (n=9 and 5, respectively) exceeded that of evergreen species (n=5 and 1, respectively), whereas the richness of deciduous and evergreen species in upland cluster and grove communities was comparable (n=5 or 6). From a taxonomic perspective, encroaching woody plants encountered in our catena-scale sampling were primarily deciduous (60% of species); but evergreen (25%) and facultative evergreen species (10%) were also well-represented. About half of the encroaching woody plants were potential N-fixers (55% of species). However, only 4 species (20%) were confirmed to have nodulation in the field. Most potential N-fixing species were deciduous; and all deciduous species had either coriaceous (6 species) or malacophyllous (6 species) leaves. Leaf texture of evergreen species (n=7) ranged from coriaceous (n=3) to malacophyllous (n=2) to sclerophyllous (n=2). Rooting depths ranged from shallow (4 species) to intermediate (7 species) to deep (3 species); but were unknown for 6 species. Rooting depths for confirmed N-fixing species were either intermediate (1 species) or deep (2 species) (and unknown for the fourth species). 

**Table 4:** Woody species functional groups and their relative basal area along the transect.

	A. Woody Species Richness						B. Woody species relative basal area (%)				
Functional Traits	Cluster (n=12)	Grove (n=13)	Woodland (n=17)	Playa (n=7)	Total (n=20)		Cluster (540)	Grove (1943)	Woodland (4,089)	Playa (315)	Total (6,987)
**Seasonal Leaf Habit ** ^***a***,***b***^											
Deciduous	6	6	9	5	13		38	46	55	60	51
Evergreen	5	5	5	1	5		48	52	20	4	31
Facultative Evergreen	1	2	2	1	2		14	2	26	37	19
**Leaf Texture ^*c*^**											
Malacohyllous	4	4	6	3**^*c*^**	7		24	1	10	23	10
Coriaceous	6	8	8	4	9		64	95	87	77	87
Sclerophyllous	2	1	2	0	2		12	4	3	0	4
Photosynthetic stem	0	0	1	1**^*b*^**	2		0	0	0	0	0
**N-fixation ^*d*^**											
N-Fixers	4	2	3	2	4		12	10	7	16	9
non N-fixers	8	11	14	5	16		88	90	93	84	91
**Rooting Depth**											
Shallow	2	2	3	2	4		42	30	17	8	22
Intermediate	4	5	6	3	7		26	33	74	76	58
Deep	3	3	3	2	3		22	18	5	16	10
Unknown	3	3	5	0	6		10	19	5	0	9

(A) Number of woody species representing various functional groups in four tree-shrub communities developing along a catena gradient in a former grassland; and (B) their relative contribution (%) to total basal area in the community (values under column headings are total basal area (cm^2^ m^-2^) in the community. See Tables 3 for species and their functional trait attributes.

^a^ *Ephedra antisyphilitica* (stem photosynthesis; woodland only) not included in count.

*b* Summer deciduous species (one only; *Zizyphus obtusifolia*) included with ‘deciduous’

*c* *Parkinsonia aculeata* has photosynthetic stems and malacophyllous leaves, so is counted in each category

*d* *Parkinsonia aculeata* is not counted as a N-fixer

From a relative basal area (BA) perspective, woody communities developing on grassland were co-dominated by evergreen (including facultative evergreen) and deciduous PFTs (Table 4). Facultative evergreen PFTs were most abundant in the two upland communities. Species with coriaceous leaves dominated all woody communities along the catena gradient (> 64% of total BA) and were most strongly expressed in upland grove and lowland woodland communities (95 and 87% of total BA, respectively). N-fixing species were clearly in the minority, contributing only 7% (woodlands) to 16 % (playas) of the total BA. Basal area in upland clusters and groves was fairly evenly partitioned among shallow, intermediate and deep-rooted PFTs, whereas PFTs with intermediate depths dominated lowland woodland and playa communities (74 and 76% of BA, respectively).

### CCA ordination

**Figure 3:**
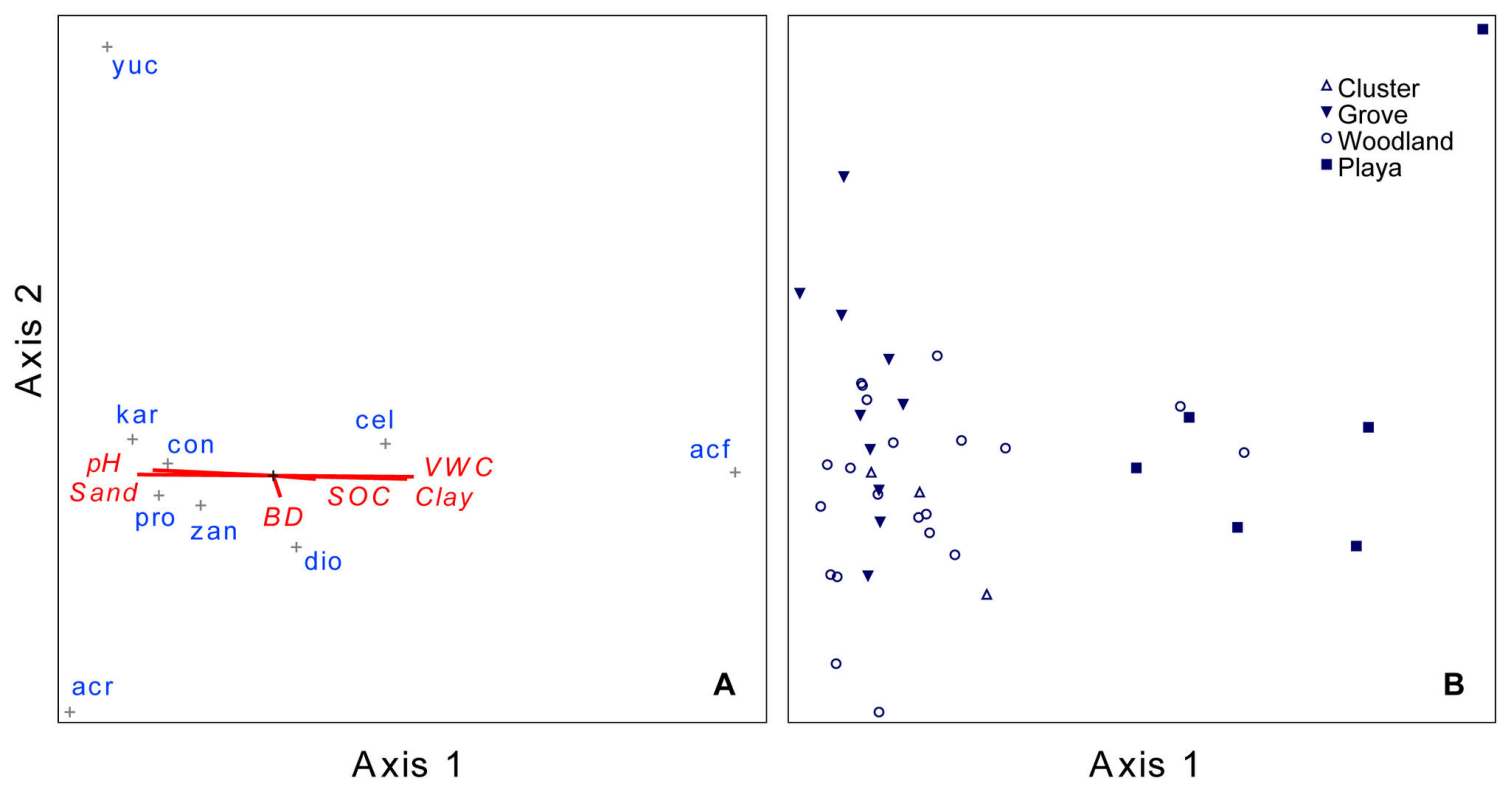
CCA Ordination (canonical correspondence analysis) results ordered in multivariate space along the first two canonical axes, separately depicting relationships among (A) tree-stature species (Table 1) and soil variables; and (B) communities in each 6 m × 6 m sample plot (symbols indicate community type) along a hill-slope transect. Species centroids (+) indicate center of distribution among sample plots for each tree species coded as follows: acf = *Acacia farnesiana*; acr = *Acacia rigidula*; cel = *Celtis pallida*; con = *Condalia hookeri*; dio *= Diospyros texana*; kar = *Karwinskia humboltiana*; pro = *Prosopis glandulosa*; zan = Zanthoxylum *fagara*. Lines are vectors indicating direction of increasing value (from center outward) for soil variables as follows: BD = bulk density; Sand and clay = soil particle percentages; SOC = soil organic carbon; TN = total nitrogen.

CCA ordination Axes I and II accounted for 18.0% and 4.4% of the total tree species inertia (3.42), respectively. The first CCA axis was strongly and positively correlated with soil clay, and negatively correlated with percent sand and pH. Tree species (Figure 3A) and communities (Figure 3B) were well-separated along this axis. There was considerable overlap among upland cluster, upland grove and lowland woodland plots. These plots were characterized by *P. glandulosa, C. hookeri and Z. fagara* and were concentrated on the higher pH, higher sand end of the axis. Plots from playa locations were quite distinct from them. Characterized by *A. farnesiana*. Playa plots occurred on the lower pH, higher clay, and higher SOC content end of Axis 1. The second CCA axis was primarily related to variation in soil BD (Figure 3A). Plots from grove and woodland communities exhibited the greatest range of variation along this axis (Figure 3B). *C. pallida* plants associated with soils having a relatively low BD and *A. rigidula* plants associated with soils with high BD defined the extremes of Axis II (Figure 3). 

**Figure 4:**
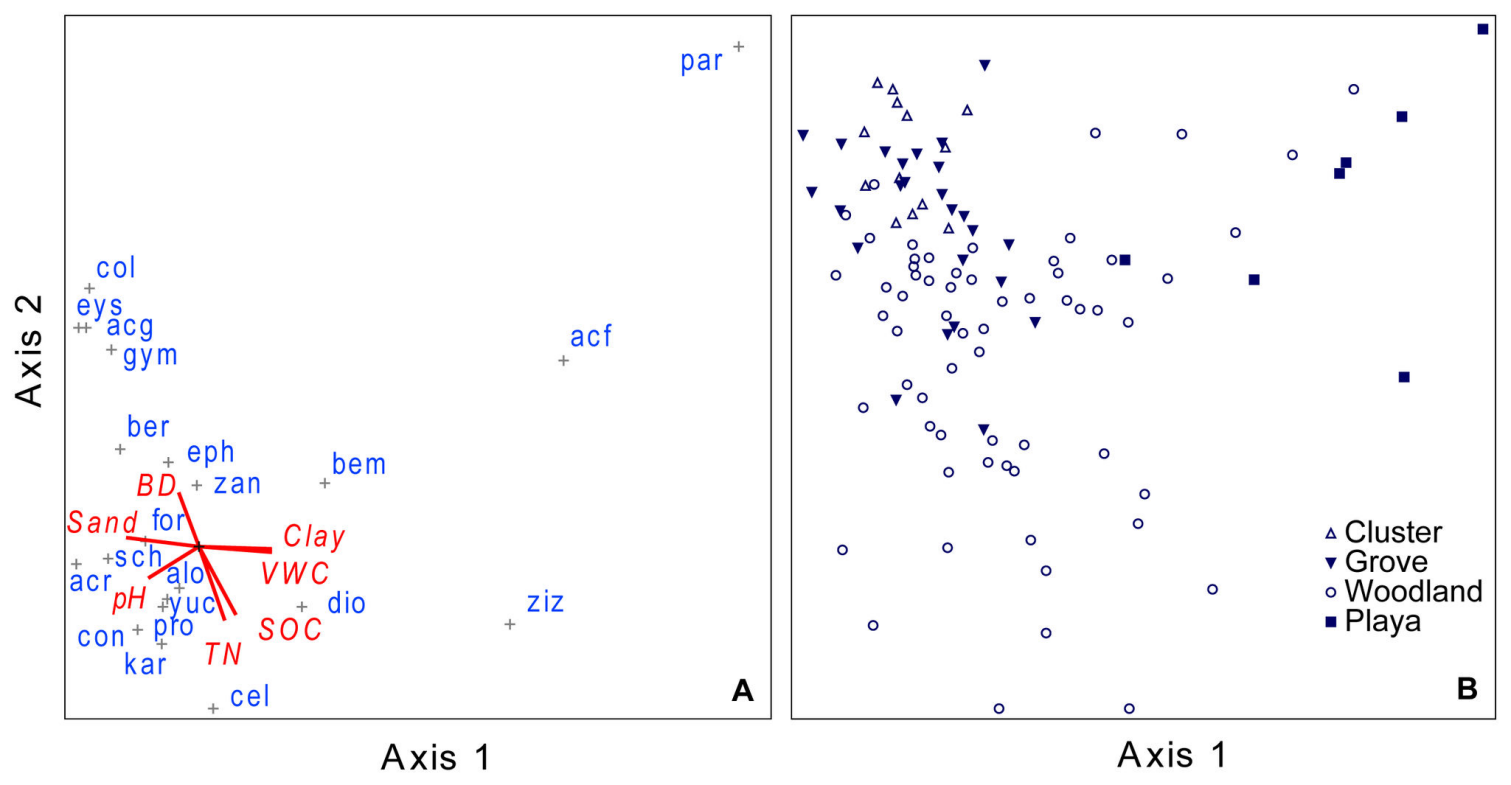
CCA Ordination (canonical correspondence analysis) results ordered in multivariate space along the first two canonical axes, separately depicting relationships among (A) shrub-stature species and soil variables (Table 1); and (B) 2 m × 2 m sample plots (symbols indicate community type) along a hill-slope transect. Species centroids (+) indicate center of distribution among sample plots for each shrub species coded as follows: acf = *Acacia farnesiana; acg = A. greggeii*; acr = *A. rigidula*; alo = *Aloysia gratissima*; bem = *Bernardia myricaefolia*; ber *= Bernardia myricaefolia*; cel *= Celtis pallida*; col = *Colubrina texensis*; con *= Condalia hookeri*; dio *= Diospyros texana*; eph = Ephedra *antisyphilitica*; eys = *Eysenhardtia texana*; for = *Forestiera angustifolia*; gym = *Gymnosperma* spp.; kar *= Karwinskia humboltiana*; par *= Parkinsonia aculeata*; pro *= Prosopis glandulosa*; sch = *Schaefferia cuneifolia*; zan = Zanthoxylum *fagara*; ziz = *Ziziphus obtusifolia*. Lines are vectors indicating direction of increasing value (from center outward) for soil variables coded as follows: BD = bulk density; Sand and clay = soil particle percentages; SOC = soil organic carbon; TN = total nitrogen.

CCA ordination of shrub-statured species exhibited greater overall variation (among more species) than tree species ordination. Total inertia was 7.85, of which 9.7% was explained by the first two axes (Axis I = 6.0%, Axis II = 3.7%). As with trees, Axis I was a synthetic gradient strongly associated with soil texture and pH (Figure 4A). The overlap among shrub cluster, grove and woodland plots observed for trees was also observed for shrubs; and, as with trees, plots from these communities were quite distinct from those in playas (Figure 4B). The centroids of most shrub species were relatively close to the plot origin, indicating a weak correlation with explanatory variables. Exceptions included *P. aculeata, A. farnesiana* and *Z. obtusifolia* in playas and some woodland plots (Figure 4). These species were positively associated with soils having higher clay content, and negatively related with soils having higher sand and pH. Axis II of the shrub ordination was mainly correlated with SOC, TN and BD (Figure 4A). Plots from woodland locations exhibited the greatest range of variation along this axis (Figure 4B); while plots from cluster and grove were more associate with higher soil BD, lower SOC and lower TN. Species like *C. pallida, C. hookeri*, and *K. humboltiana* were most strongly associated with soils having higher SOC and TN, whereas other shrub species (e.g., *C. texensis, E. texana, A. greggeii*) were more strongly associated with soils having relatively high BD. 

### Variation partitioning

**Figure 5:**
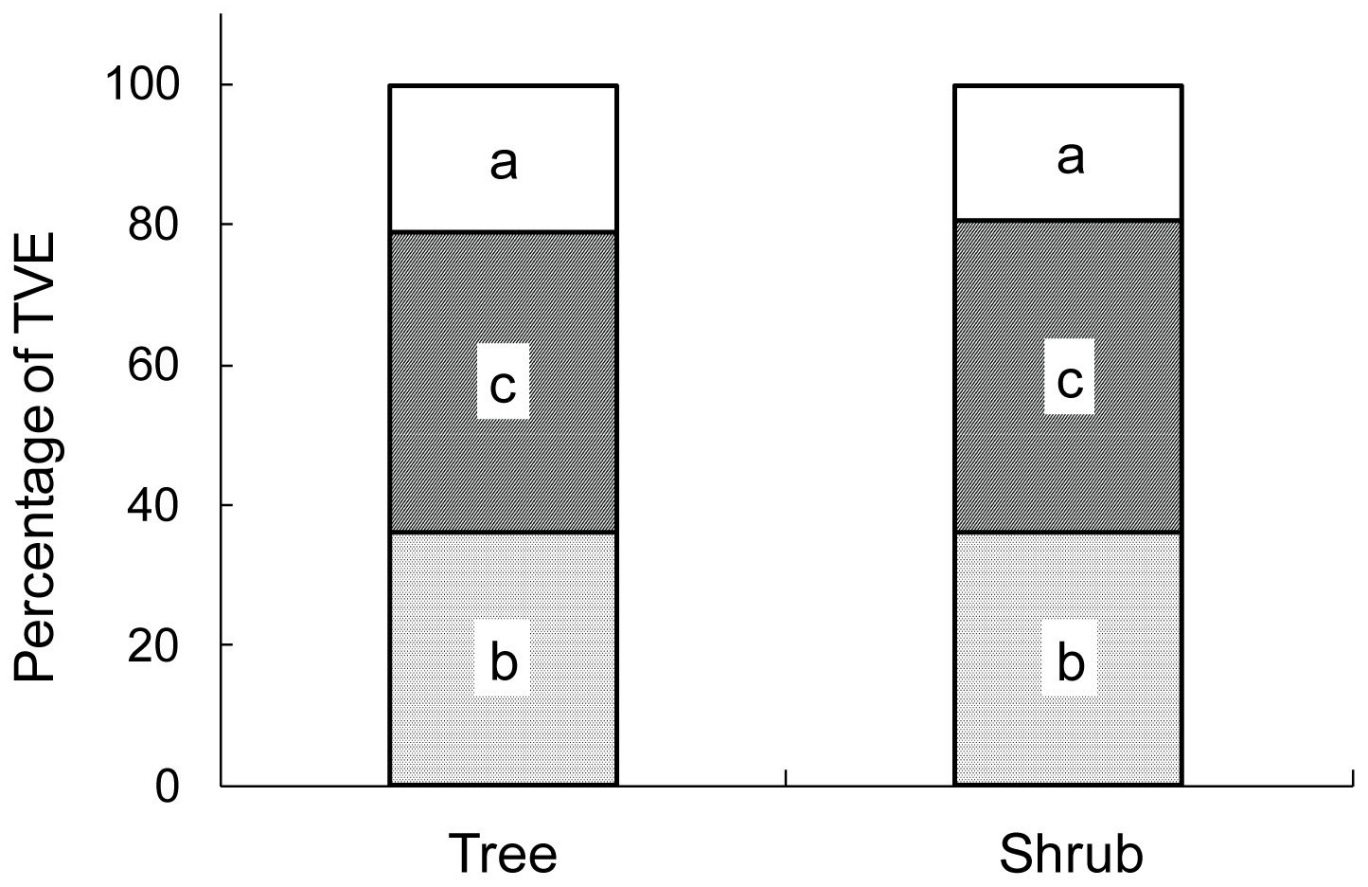
Variation decomposition shown as a percentage of total variation explained (TVE) for separate analyses of tree (Figure 3) and shrub (Figure 4) plots, that were uniquely related to variable groupings that included (a) soil organic carbon and total nitrogen, (b) % sand and % clay; or (c) an equal sharing by a and b.

Soil texture (% sand, % clay) accounted for the largest percentage of the unique total variation explained (TVE) for both tree (27.4%) and shrub (22.8%) species (Figure 5). A smaller percentage of the unique TVE was explained by SOC and TN (14.7% for trees and 18.7% for shrubs; Figure 5). However, the largest portion of the explained variation in both tree (57.9%) and shrub (58.5%) species distribution was that shared between both groups of soil variables (i.e., each group of variables redundantly explained the residual percentage of the TVE after the variation unique to each group was accounted for).

## Discussion

Isotopic evidence indicates the discrete cluster, grove and woodland communities at this site have all developed on what were grassland communities; and lines of evidence indicate these woodland communities are relatively recent (< 60-100 y of age)[1,46]. Thus, the discrete upland cluster, grove and drainage woodland communities represent examples of topoedaphic mediation of woody plant encroachment and community development. In this study we sought to determine if there were fundamental differences in the species and PFT composition of these communities that might shed light on how the shrub encroachment process plays out on upland and lowland portions of grassland landscapes. Our assessments of species and PFT composition and abundance were conducted across a local landscape-scale gradient. As such, they are not confounded by differences in climate, weather or land use history that can occur when comparing species abundance and community structure at multiple sites within a region or across bioclimatic zones. Here we only selected several categorical functional traits to define different PFTs, which we believe represent a wide range of functional traits that are important. However, including more detailed quantitative traits such as seed size or leaf traits may make our case stronger by providing other important aspects of the community assemblage.

Soil properties differed substantially between upland shrub cluster and grove communities [1] and along upland-to-lowland elevation gradients (Figure 1). Even so, upland cluster and grove communities were highly similar to each other and to lowland woodland communities (Figures 3a and 4a). Playa savanna communities, which are subject to periods of inundation that can cause woody plant mortality following heavy rains [34,35], were quite distinct from these, more so with the tree component than with the shrub component. However, with the exception of *P. aculeata*, the woody species and PFTs in playa communities were also present and typically common in cluster, grove, and woodland communities (Tables 2, 3, and 4). Furthermore, the extremes of the Axis 1 ordination gradient for both tree- and shrub-statured woody plants were characterized by similar PFTs (deciduous, N_2_-fixing, intermediate-to-deep rooting species; Figures 3, 4). Thus, we found little evidence to suggest that the major encroaching tree/shrub species or the PFTs they represent had strong preferences for or were uniquely confined to a given landscape location. Furthermore, it appears that patterns of both species presence/absence and their abundance along the hill-slope gradient were primarily a function of differences in intrinsic soil physical properties (e.g., texture and bulk density) and secondarily related to SOC and TN (Figure 5). 

The woody communities on this former grassland site are relatively young (< 60-100 y of age) and may still be developing. Their species and PFT composition in the future may, to a large extent, depend upon the extent to which the present-day dominants are represented in the understory shrub layer. Overstory Prosopis plants (arborescent, deciduous, and N_2_-fixing) have been dying in cluster communities [1] as was evidenced by its absence in our sampling (Table 2). A. farnesiana, representing the same PFT as P. glandulosa, also occurred in clusters, but was not represented in the shrub layer (Table 3) and hence may not persist in the community. However other N_2_-fixing woody species occur in the shrub layer, so this PFT could still be represented in future vegetation states even with the demise of the current N-fixing PFTs in the tree layer. Prosopis overstory plants were doing well in the other communities (Table 2); and, with the exception of playa communities, was well-represented in the shrub class (Table 3). This suggests that overstory trees of this PFT will be replaced when they die. These observations centered around N_2_-fixing, deciduous PFTs are consistent with ordination results (Figures 3 and 4) and suggest two things: (i) significant changes in the species composition of each of these woody plant communities may be forthcoming; and (ii) species-specific rather than PFT-specific differences are driving the compositional dynamics of the woody communities developing on grassland. In other words, while some species may eventually be lost, the PFTs they represent will not be.

The N_2_-fixing PFT was a small component of the pool of encroaching woody plant species at our subtropical site from both a species richness and dominance (relative basal area) perspective (Table 4A and B). This is consistent with observations in Africa [47], despite the fact that leguminous trees are often regarded as a key component of African savannas [48]. However, a small contribution of N_2_-fixing shrubs to the species pool or total basal area, does not necessarily reflect their ecological importance. Indeed, early investigations at our study site suggested that non-N-fixing shrubs colonize soils that have been enriched in organic matter and nitrogen by N-fixing PFTs [49]. 

Rooting depth is an important functional trait in savanna ecosystems because niche separation of soil water use at different depth was believed to be an importance mechanism to tree-grass coexistence [49,50]. In our study site, as woody invasion is initiated by the establishment of *P. glandulosa* (deep rooted) on this former grassland, *P. glandulosa* can quickly grow roots deeper than the zone utilized by grasses, which enhanced their survival during the earlier stage of their life history [51]. Later in developed woody communities, hydraulic lift by *P. glandulosa* is an important mechanism of vertical water redistribution. However, the hydraulic lift is temporal dynamic and its effect on understory shrubs varies [31]. Overall, species interactions between overstory trees and understory shrubs are competitive and may cause the demise of many *P. glandulosa* trees [30,52]. Our observations of rooting depth PFTs in woody communities along the topoedaphic gradient (Table 4) suggest that the PFTs will very likely persist even though the dominance of individual species may vary in the future. 

Relative basal area of evergreen and deciduous woody species are relatively stable along the topoedphic gradient (Table 4). Facultative evergreen functional type is more abundant in woodland and playa than the evergreen PFT, which is probably due to the associated soil moisture conditions along the edaphic gradient. It is very likely that deciduous and evergreen (including facultative) PFTs will persist in the communities with relatively stable percentages. However, more quantitative ecophysiological leaf traits (such as photosynthesis rate and specific leaf area) may be more indicative on how the functional traits may change over time, due to the differences between native and invasive species [21,53]. 

The number of woody species in shrub cluster communities and their patterns of occurrence at this site are strongly related to the size/age of the overstory trees [54]. Soils and microclimate change as the woody communities develop [1,55], and shrub species within these communities differ with respect to leaf longevity, specific leaf area, texture, and N content [21], daily and seasonal patterns of photosynthesis and water relations [30,32], functional rooting depths [31,52], and nitrogen responses [22,37]. These observations suggest a potential basis for differentiation among PFTs as woody plant communities develop on grassland. However, when confounding differences in abundance among shrub species are accounted for and compared against neutral predictions, random processes account for nearly all patterns of species and PFT occurrence [56]. This is consistent with observations that the classification of plants into functional groups based on leaf traits and N_2_-fixation potential are not necessarily reliable indicators of ecosystem processes related to resource use [21] or decomposition [57]. 

While there were numerous N_2_-fixing PFTs at our site, *P. glandulosa* was clearly the most aggressive initial invader of the historical grasslands [54]. This may reflect the fact that this species (i) is widely and effectively dispersed by grazing livestock [58]; and (ii) develops a root system that accesses water beyond the rooting zone of grasses very early in its life cycle [59]. Thus, traits related to seed dispersal and early taproot elongation may be more important in explaining the success of this species than N_2_-fixation. In addition, most of the other non N_2_-fixing woody PFTs at this site are bird-dispersed; and the establishment of livestock-dispersed *P. glandulosa* provides a perching structure attracting birds disseminating seeds of other woody PFTs [1]. This suggests that modification of soils by *P. glandulosa* plants colonizing grasslands may therefore be a secondary or coincidental factor influencing the encroachment of other woody PFTs – a conjecture consistent with our finding that patterns of species and PFT presence/absence and abundance were primarily a function of differences in intrinsic soil physical properties and secondarily related to modifications of SOC and TN by early-establishing shrubs (Figure 5).

Modification of soil properties beneath woody plant canopies is well-documented for this site [9,23,60] and for many other arid and semi-arid ecosystems undergoing increases in woody plant abundance (e.g., 61-63). Although several studies have implicated changes in soil C and N as a driver of plant distribution and species turnover [64-67], our results suggest that in the context of woody plant encroachment into grasslands, these effects are secondary to the influences of landform or geomorphic variables. However, in drier sites, shrub-soil feedbacks could play a more important role in driving woody encroachment. Variance partitioning results indicate that changes in SOC and soil N are likely to influence productivity and nutrient availability and may, over longer time-frames, feed back to influence PFT distributions. The two groups of variables also shared ~ 58% of TVE. This shared variation may reflect important interrelationships between relatively slow- and fast-changing edaphic properties or that both are related to another unmeasured variable. Both groups of variables exhibited similar spatial trends along the hill-slope, with higher values of SOC, TN, and clay in drainage woodlands than that in uplands (Figure 2). This suggests that correlations between these two groups probably contributed a significant portion of the shared variation. Dynamic simulations indicate that shrub-induced changes in SOC and TN will continue to increase for decades to come on this site [68]. However, while woody species composition may change, our data provide no basis for expecting that some woody PFTs will fare better than others.

The results from our study are consistent with assertions of random community assembly [56] in that woody species on this site were, for the most part, ubiquitously distributed along the topoedaphic gradient with its wide-ranging soil properties. Shrub species and the PFTs they represent in the Argentinian Caldenal, which is physiognomically similar to the Tamaulipan thornscrub of our Southern Great Plains study site, are also widely distributed across broad environmental gradients [69]. Thus, although the early establishment of *Prosopis* (a deep-rooted, N_2_-fixing, deciduous arborescent) appears to be important in initiating the transition from grassland to shrubland across the catena gradient at this site, our survey gives little reason to expect that the shrubland communities that subsequently develop are following any particular or predictable assembly rules that might consistently reflect PFT responses to changes in microclimate and soils occurring in the transition from grassland to shrubland or woodland. The lack of strong patterns in woody species or PFT occurrence within the four woody plant communities developing on contrasting topoedaphic settings may reflect the fact that PFTs can exhibit considerable convergence in resource use [70] and that there are many viable strategies for coping with given sets of environmental conditions. In the context of the physiognomic conversion of grasslands to woodlands, the ubiquitous distribution of shrub and arborescent species and PFTs on this site suggests that woody plants, and the diverse growth forms they include, are well-suited to a broad range of grassland topoedaphic settings. 

## References

[1] Archer S (1995) Tree-grass dynamics in a *Prosopis*-thornscrub savanna parkland - reconstructing the past and predicting the future. EcoScience 2: 83-99.

[2] Van Auken OW (2000) Shrub invasions of North American semiarid grasslands. Annual Review of Ecology and Systematics 31: 197-215. doi:10.1146/annurev.ecolsys.31.1.197.

[3] Naito AT, Cairns DM (2011) Patterns and processes of global shrub expansion. Progress in Physical Geography 35: 423-442. doi:10.1177/0309133311403538.

[4] Knapp AK, Briggs JM, Collins SL, Archer SR, Bret-Harte MS et al. (2008) Shrub encroachment in North American grasslands: shifts in growth form dominance rapidly alters control of ecosystem carbon inputs. Global Change Biology 14: 615-623. doi:10.1111/j.1365-2486.2007.01512.x.

[5] Biederman LA, Boutton TW (2009) Biodiversity and trophic structure of soil nematode communities are altered following woody plant invasion of grassland. Soil Biology and Biochemistry 41: 1943-1950. doi:10.1016/j.soilbio.2009.06.019.

[6] Archer S (2010) Rangeland conservation and shrub encroachment: new perspectives on an old problem. In: Toit Kock R Deutsch J. Rangelands or Wildlands? Livestock and wildlife in semi-arid ecosystems. Oxford, England: Backwell Publishing . pp. 53-97

[7] Dickie IA, Yeates GW, St John MG, Stevenson BA, Scott JT et al. (2011) Ecosystem service and biodiversity trade-offs in two woody successions. Journal of Applied Ecology 48: 926-934. doi:10.1111/j.1365-2664.2011.01980.x.

[8] Eldridge DJ, Bowker MA, Maestre FT, Roger E, Reynolds JF et al. (2011) Impacts of shrub encroachment on ecosystem structure and functioning: towards a global synthesis. Ecol Lett 14: 709-722. doi:10.1111/j.1461-0248.2011.01630.x. PubMed: 21592276. 21592276 PMC3563963

[9] Boutton TW, Liao JD, Filley TR, Archer SR (2009) Belowground carbon storage and dynamics accompanying woody plant encroachment in a subtropical savanna. In: Lal R Follett R Soil carbon sequestration and the greenhouse effect. Madison, WI: Soil Science Society of America pp. 181-205.

[10] Houghton RA, Hackler JL, Lawrence KT (1999) The US carbon budget: contributions from land-use change. Science 285: 574-578. doi:10.1126/science.285.5427.574. PubMed: 10417385. 10417385

[11] Barger NN, Archer SR, Campbell JL, Huang CY, Morton JA, et al. (2011) Woody plant proliferation in North American drylands: A synthesis of impacts on ecosystem carbon balance. Journal of Geophysical Research - Biogeosciences 116: G00K07, doi:10.1029/2010JG001506.

[12] Asner GP, Archer S (2009) Livestock and the global carbon cycle. In: Steinfeld H Mooney H Schneider F Neville L Livestock in a changing landscape: drivers, consequences, and responses. Washington, D.C.: Island Press pp. 69-82.

[13] Schlesinger WH, Reynolds JF, Cunningham GL, Huenneke LF, Jarrell WM et al. (1990) Biological feedbacks in global desertification. Science 247: 1043-1048. doi:10.1126/science.247.4946.1043. PubMed: 17800060. 17800060

[14] Tape K, Sturm M, Racine C (2006) The evidence for shrub expansion in Northern Alaska and the Pan-Arctic. Global Change Biology 12: 686-702. doi:10.1111/j.1365-2486.2006.01128.x.

[15] Higgins SI, Bond WJ, Combrink H, Craine JM, February EC et al. (2012) Which traits determine shifts in the abundance of tree species in a fire-prone savanna? Journal of Ecology 100: 1400-1410. doi:10.1111/j.1365-2745.2012.02026.x.

[16] Wakeling JL, Staver AC, Bond WJ (2011) Simply the best: the transition of savanna saplings to trees. Oikos 120: 1448-1451. doi:10.1111/j.1600-0706.2011.19957.x.

[17] Ratajczak Z, Nippert JB, Hartman JC, Ocheltree TW (2011) Positive feedbacks amplify rates of woody encroachment in mesic tallgrass prairie. Ecosphere 2: art121

[18] McLendon T (1991) Preliminary description of the vegetation of South Texas exclusive of coastal saline zones. Texas Journal of Science 43: 13-32.

[19] Coffey CR (1986) A floristic study of the La Copita Reaserch Area in Jim Wells County. Texas Department of Rangeland Ecology and Management, Texas A&M University.

[20] Watts SE (1993) Rooting patterns of co-occurring woody plants on contrasting soils in a subtropical savanna. College Station,TX: Texas A&M University.

[21] Nelson JA, Barnes PW, Archer S (2002) Leaf demography and growth responses to altered resource availability in woody plants of contrasting leaf habit in a subtropical savanna. Plant Ecology 160: 193-205. doi:10.1023/A:1015828604444.

[22] Zitzer SF, Archer SR, Boutton TW (1996) Spatial variability in the potential for symbiotic N-2 fixation by woody plants in a subtropical savanna ecosystem. Journal of Applied Ecology 33: 1125-1136. doi:10.2307/2404692.

[23] Archer S, Boutton TW, Hibbard KA (2001) Trees in grasslands: biogeochemical consequences of woody plant expansion. In: Schulze ED Harrison SP Heimann M Holland EA Lloyd J Global biogeochemical cycles in the climate systems. San Diego: Academic Press pp. 115-137.

[24] Van Auken OW (2009) Causes and consequences of woody plant encroachment into western North American grasslands. J Environ Manage 90: 2931-2942. doi:10.1016/j.jenvman.2009.04.023. PubMed: 19501450. 19501450

[25] Browning DM, Archer SR, Asner GP, McClaran MP, Wessman CA (2008) Woody plants in grasslands: Post-encroachment stand dynamics. Ecol Appl 18: 928-944. doi:10.1890/07-1559.1. PubMed: 18536253. 18536253

[26] Weltzin JF, Archer S, Heitschmidt RK (1997) Small-mammal regulation of vegetation structure in a temperate Savanna. Ecology 78: 751-763. Available online at: doi:10.1890/0012-9658(1997)078[0751:SMROVS]2.0.CO;2

[27] Blair WF (1950) The biotic provinces of Texas. Journal of Science 2: 93-117.

[28] Reid N, Smith DMS, Beyermunzel P, Marroquin J (1990) Floristic and structural variation in the Tamaulipan thornscrub, Northeastern Mexico. Journal of Vegetation Science 1: 529-538. doi:10.2307/3235787.

[29] Barnes PW, Archer S (1996) Influence of an overstorey tree (*Prosopis* *glandulosa*) on associated shrubs in a savanna parkland: implications for patch dynamics. Oecologia 105: 493-500. doi:10.1007/BF00330012. 28307142

[30] Barnes PW, Archer S (1999) Tree-shrub interactions in a subtropical savanna parkland: competition or facilitation? Journal of Vegetation Science 10: 525-536. doi:10.2307/3237187.

[31] Zou CB, Barnes PW, Archer S, McMurtry CR (2005) Soil moisture redistribution as a mechanism of facilitation in Savanna tree-shrub clusters. Oecologia 145: 32-40. doi:10.1007/s00442-005-0110-8. PubMed: 15942764. 15942764

[32] Bai E, Boutton TW, Liu F, Ben Wu X, Archer SR (2008) Variation in woody plant delta C-13 along a topoedaphic gradient in a subtropical savanna parkland. Oecologia 156: 479-489. doi:10.1007/s00442-008-1003-4. PubMed: 18327619. 18327619

[33] Bai E, Boutton TW, Liu F, Ben Wu X, Archer SR (2012) Spatial patterns of soil δ13C reveal grassland-to-woodland successional processes. Organic Geochemistry 42: 1512-1518. doi:10.1016/j.orggeochem.2010.11.004.

[34] Parker AL, Owens PR, Libbohova Z, Wu XB, Wilding L, Archer SR (2010) Ecohydrology of playa-wetland ecosystems: a landscape-scale perspective. Journal of Arid Environments: 1487-1493.

[35] Scifres C, Mutz JL (1975) Secordary succession following extended inundation of Texas coastal rangeland. Journal of Range Management 28: 279-282. doi:10.2307/3897776.

[36] Everitt JH, Drawe DL, Lonard RI (2002) Trees, shrubs, and cacti of south Texas. Texas Tech University Press. 264 pp.

[37] Boutton TW, Archer SR, Midwood AJ (1999) Stable isotopes in ecosystem science: structure, function and dynamics of a subtropical savanna. Rapid Commun Mass Spectrom 13: 1263-1277. doi:10.1002/(SICI)1097-0231(19990715)13:13. PubMed: 10407309. 10407309

[38] Soil Survey Staff (2004) Soil Survey Laboratory methods manual. Soil Survey Investigations Report No. 42

[39] TerBraak CJF (1986) Canonical Correspondence-Analysis - a New Eigenvector Technique for Multivariate Direct Gradient Analysis. Ecology 67: 1167-1179. doi:10.2307/1938672.

[40] McCune B, Mefford MJ (1999) PC-ORD Multivariate Analysis of Ecological Data, version 4: MjM Software Design, Gleneden Beach, Oregon, USA

[41] Økland RH (1999) On the variation explained by ordination and constrained ordination axes. Journal of Vegetation Science 10: 131-136. doi:10.2307/3237168.

[42] Qian H, Klinka K, Økland RH, Krestov P, Kayahara GJ (2003) Understorey vegetation in boreal *Picea* *mariana* and *Populus* *tremuloides* stands in British Columbia. Journal of Vegetation Science 14: 173-184. Available online at: doi:10.1658/1100-9233(2003)014[0173:UVIBPM]2.0.CO;2

[43] Økland RH, Eilertsen O (1994) Canonical correspondence-analysis with variation partitioning - some comments and an application. Journal of Vegetation Science 5: 117-126. doi:10.2307/3235645.

[44] Borcard D, Legendre P, Drapeau P (1992) Partialling out the spatial component of ecological variation. Ecology 73: 1045-1055. doi:10.2307/1940179.

[45] Økland RH (2003) Partitioning the variation in a plot-by-species data matrix that is related to n sets of explanatory variables. Journal of Vegetation Science 14: 693-700. Available online at: doi:10.1658/1100-9233(2003)014[0693:PTVIAP]2.0.CO;2

[46] Boutton TW, Archer SR, Midwood AJ, Zitzer SF, Bol R (1998) Delta C-13 values of soil organic carbon and their use in documenting vegetation change in a subtropical savanna ecosystem. Geoderma 82: 5-41. doi:10.1016/S0016-7061(97)00095-5.

[47] Wigley BJ, Bond WJ, Hoffman MT (2010) Thicket expansion in a South African savanna under divergent land use: local vs. global drivers? Global Change Biology 16: 964-976. doi:10.1111/j.1365-2486.2009.02030.x.

[48] Midgley JJ, Bond WJ (2001) A synthesis of the demography of African acacias. Journal of Tropical Ecology 17: 871-886.

[49] Scholes RJ, Archer SR (1997) Tree-grass interactions in savannas. Annual Review of Ecology and Systematics 28: 517-544. doi:10.1146/annurev.ecolsys.28.1.517.

[50] Sankaran M, Ratnam J, Hanan NP (2004) Tree-grass coexistence in savannas revisited - insights from an examination of assumptions and mechanisms invoked in existing models. Ecology Letters 7: 480-490. doi:10.1111/j.1461-0248.2004.00596.x.

[51] Brown JR, Archer S (1990) Water relations of a perennial grass and seedling vs adult woody-plants in a subtropical savanna, Texas. Oikos 57: 366-374. doi:10.2307/3565966.

[52] Midwood AJ, Boutton TW, Archer SR, Watts SE (1998) Water use by woody plants on contrasting soils in a savanna parkland: assessment with delta H-2 and delta O-18. Plant and Soil 205: 13-24. doi:10.1023/A:1004355423241.

[53] Liao C, Peng R, Luo Y, Zhou X, Wu X et al. (2008) Altered ecosystem carbon and nitrogen cycles by plant invasion: a meta-analysis. New Phytol 177: 706-714. doi:10.1111/j.1469-8137.2007.02290.x. PubMed: 18042198. 18042198

[54] Archer S, Scifres C, Bassham CR, Maggio R (1988) Autogenic succession in a sub-tropical savanna - conversion of grassland to thorn woodland. Ecological Monographs 58: 111-127. doi:10.2307/1942463.

[55] Hibbard KA, Archer S, Schimel DS, Valentine DW (2001) Biogeochemical changes accompanying woody plant encroachment in a subtropical savanna. Ecology 82: 1999-2011. Available online at: doi:10.1890/0012-9658(2001)082[1999:BCAWPE]2.0.CO;2

[56] Stokes CJ, Archer SR (2010) Niche differentiation and neutral theory: an integrated perspective on shrub assemblages in a parkland savanna. Ecology 91: 1152-1162. doi:10.1890/08-1105.1. PubMed: 20462129. 20462129

[57] Kurokawa H, Peltzer DA, Wardle DA (2010) Plant traits, leaf palatability and litter decomposability for co-occurring woody species differing in invasion status and nitrogen fixation ability. Functional Ecology 24: 513-523. doi:10.1111/j.1365-2435.2009.01676.x.

[58] Brown JR, Archer S (1987) Woody plant seed dispersal and gap formation in a North American subtropical savanna woodland: the role of domestic herbivores. Vegetatio 73: 73-80.

[59] Jurena PN, Archer S (2003) Woody plant establishment and spatial heterogeneity in grasslands. Ecology 84: 907-919. Available online at: doi:10.1890/0012-9658(2003)084[0907:WPEASH]2.0.CO;2

[60] Boutton TW, Liao JD (2010) Changes in soil nitrogen storage and δ^15^N with woody plant encroachment in a subtropical savanna parkland landscape. Journal of Geophysical Research - Biogeosciences 115: G03019, doi:03010.01029/02009JG001184.

[61] Vetaas OR (1992) Micro-site effects of trees and shrubs in dry savannas. Journal of Vegetation Science 3: 337-344. doi:10.2307/3235758.

[62] Rossi BE, Villagra PE (2003) Effects of *Prosopis* *flexuosa* on soil properties and the spatial pattern of understorey species in arid Argentina. Journal of Vegetation Science 14: 543-550. doi:10.1111/j.1654-1103.2003.tb02181.x.

[63] Garcia C, Roldan A, Hernandez T (2005) Ability of different plant species to promote microbiological processes in semiarid soil. Geoderma 124: 193-202. doi:10.1016/j.geoderma.2004.04.013.

[64] Burke IC, Lauenroth WK, Vinton MA, Hook PB, Kelly RH et al. (1998) Plant-soil interactions in temperate grasslands. Biogeochemistry 42: 121-143. doi:10.1023/A:1005987807596.

[65] Schlesinger WH, Pilmanis AM (1998) Plant-soil interactions in deserts. Biogeochemistry 42: 169-187. doi:10.1023/A:1005939924434.

[66] Van Breemen N, Finzi AC (1998) Plant-soil interactions: ecological aspects and evolutionary implications. Biogeochemistry 42: 1-19. doi:10.1023/A:1005962124317.

[67] Laungani R, Knops JMH (2009) Species-driven changes in nitrogen cycling can provide a mechanism for plant invasions. Proceedings of the National Academy of Sciences of the USA 106: 12400-12405. doi:10.1073/pnas.0900921106. PubMed: 19592506. 19592506 PMC2718360

[68] Hibbard KA, Schimel DS, Archer S, Ojima DS, Parton W (2003) Grassland to woodland transitions: integrating changes in landscape structure and biogeochemistry. Ecological Applications 13: 911-926. Available online at: doi:10.1890/1051-0761(2003)13[911:GTWTIC]2.0.CO;2

[69] Bóo RM, Peláez DV (1991) Ordenamiento y clasificación de la vegetación en un área del sur del Distrito del Caldén. Boletín de la Sociedad Argentina de Botánica 27: 135–141.

[70] O'Grady AP, Cook PG, Eamus D, Duguid A, Wischusen JD et al. (2009) Convergence of tree water use within an arid-zone woodland. Oecologia 160: 643-655. doi:10.1007/s00442-009-1332-y. PubMed: 19333625. 19333625

